# Effect of Frozen Storage on Molecular Weight, Size Distribution and Conformation of Gluten by SAXS and SEC-MALLS

**DOI:** 10.3390/molecules17067169

**Published:** 2012-06-12

**Authors:** Lei Zhao, Lin Li, Guo-Qin Liu, Xing-Xun Liu, Bing Li

**Affiliations:** 1 College of Light Industry and Food Sciences, South China University of Technology, Guangzhou 510640, China; 2 Guangdong Province Key Laboratory for Green Processing of Natural Products and Product Safety, Guangzhou 510640, China

**Keywords:** gluten, frozen storage, SEC-MALLS, SAXS

## Abstract

In this study, the effects of frozen (−18 °C) storage time on molecular weight, size distribution, conformation, free amino groups and free sulfhydryl groups of gluten were studied by small-angle X-ray scattering (SAXS), multi-angle laser light scattering (MALLS) in conjunction with a size exclusion chromatography (SEC) and spectrophotometrically. The results showed that the gluten dissolved in 50 mM acetic acid appeared to be similar to quasi-spherical of the chain conformation and the slope of theconformation plot decreased during the storage. Both the molecular weight and radius of gyration of the frozen gluten decreased with the storage time showing a depolymerization in the high molecular weight fraction of gluten (10^5^ Da ~ 10^9^ Da). Therefore, at constant molecular weight the change of the chain conformation did not show a clear correlation with the storage time. The free amino groups content changed little and the free sulfhydryl groups content of the gluten increased from 9.8 μmol/g for the control to 12.87 μmol/g for 120-day-stored gluten, indicating that the water redistribution and ice recrystallization lead to the breakage of the disulphide bonds and may be one of the reasons for the depolymerization of gluten polymer.

## 1. Introduction

The frozen food market has grown steadily in recent years due to the convenience and high quality of frozen foods. With the demand and market opportunities for frozen wheat-based products, frozen dough is an attractive alternative of the unfrozen version. It was reported that the molecular weight (*M_w_*) and distribution of the wheat gluten protein complexes are related with dough texture and baking properties. Southan and MacRitchie proved that the properties of dough such as tensile strength were determined by a fraction of polymer with *M_w_* above a certain critical value (1 × 10^6^ Da) and the molecular weight distribution (MWD) of this fraction [1]. 

Using the SDS gel electrophoresis technique Kennedy reported a considerable increase in the number of lower molecular weight oligomers which presumably arose from depolymerization of glutenin, resulting in weakening the gluten protein structure [2]. *M_w_* of the gluten is more important for the quality of the final product during the frozen storage. Because of the connection of *M_w_* to functionality, attention has been focused on the *M_w_* of gluten proteins in frozen foods during the frozen storage process. A decrease in the amount of glutenin subunits of high molecular weight during storage at −18 °C was also reported [3]. With the development of the corresponding instrumentation new methods have recently become widely used to detect the molecular weight and distribution, e.g., Kuktaite *et al*. [4] and Gupta *et al*. [5] used size-exclusion high-performance liquid chromatography (SE-HPLC) to investigate the change of size distribution in the gluten during dough processing.

Nowadays, small angle X-ray scattering (SAXS) and multiangle laser light scattering (MALLS) in conjunction with a size exclusion chromatography (SEC) have been used to characterize macromolecule structure changes in solution [6]. Watanabe *et al*. reported that the SEC-SAXS technique would be useful for the determination of the radius of gyration (*R_g_*) value and *M_w_* of the separated protein molecules with dimensions of 1–10 nm in solution [7]. Egelhaaf *et al*. stated that the structure of the central repetitive domain (*dB1* and *dB4*) of high *M_w_* wheat gluten proteins was cylindrical and flexible [8].

The SEC-MALLS is also a quick, reliable technique for determining the *M_w_* and *R_g_* of macromolecules [9]. Bean and Lookhart characterized wheat protein using the SEC-MALLS technique and found that the *M_w_* of the SDS-insoluble protein was 8.1 × 10^7^ Da [10]. Mendichi *et al*. used the SEC-MALLS technique and reported that the *M_w_* of *Cheyenne* and *Chinese Spring* glutenin polymers was a complex mixture of high molecular weight (HMW) and low molecular weight (LMW) fractions, and the relative amount of HMW fractions in the *Chinese Spring* polymer was considerably lower than the *Cheyenne* polymer (19.2% and 47.1%, respectively) [11]. Substantially, the SAXS and SEC-MALLS technique is shown to be a useful tool for characterizing gluten protein polymers when choosing the appropriate experiment condition. In this work, the objective was to further investigate the effect of prolonged frozen storage on the solution conformation, *M_w_* and size distribution of the gluten by the SAXS and SEC-MALLS techniques.

## 2. Results and Discussion

### 2.1. SAXS Analysis

**Figure 1:**
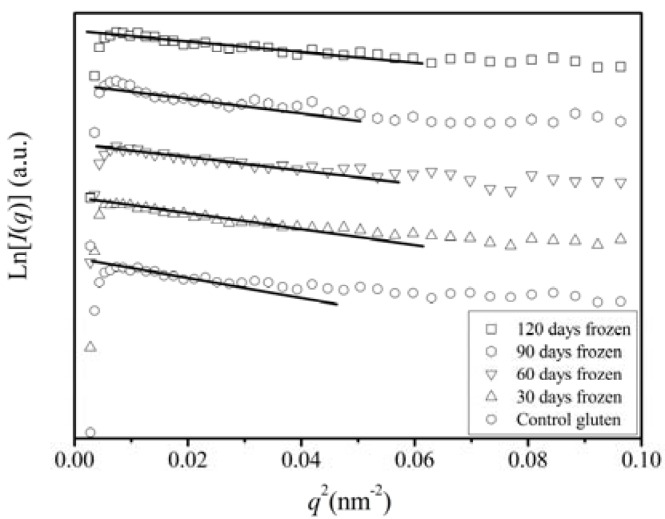
Guinier plots scattering curves from various frozen storage time states of gluten.

The SAXS technique is shown to be a useful tool for investigating solution structure of proteins. The scattering data for the gluten samples frozen stored for different time were plotted as ln (*I*) against *q*^2^ (Figure 1), where *I* is the corrected scattering intensity and *q* = (4π / λ) sin (*θ* / 2) is the scattering vector. The Guinier plots (*q*^2^ *vs*. ln*I*) of the measured SAXS data revealed various conformational forms of the gluten proteins. The low *q*-region of the scattering curves was approximated by *I*(*q*) = *I*(*0*) exp(−*q*^2^*R*_g_^2^/3) where *I*(0) is the scattering intensity at the zero scattering angle and *R*_g(SAXS)_ is the radius of gyration determined by SAXS. The plot of ln (*I*(*q*)) *vs.* *q*^2^ yields a straight line, the slop of which gives the *R*_g(SAXS)_ value. All the curves in the region of *q*^2^ < 0.04 nm^−2^ were approximated by a straight line, and the low-*q* limit of the Guinier rule (*qR_g(SAXS)_* < 1) was *q*^2^ < 0.007–0.024 nm^−2^. In the Guinier plot, each scattering curve was well fitted to a straight line, indicating that the gluten proteins were relatively homogeneous in terms of the configuration.

The values of *R_g(SAXS)_* for all gluten samples frozen stored for different times, which were estimated from the slope value of the regression line within the Guinier region, are listed in Table 1. From Table 1, it can been seen that the *R_g(SAXS)_* for frozen gluten proteins obviously decreased with increasing storage time. The *R_g(SAXS)_* of the frozen control gluten (*R_g(SAXS)_* = 12.84 nm) was more than two-fold larger than that of the frozen gluten stored for 120 days (*R_g(SAXS)_* = 6.21 nm). The decrease in radius of gyration implied the higher compactness of a polymer molecule at the same molecular weight or a decrease in molecule weight for the polymer molecules. In the case of the frozen gluten macropolymers, it was more likely that the molecule weight of gluten was decreased due to the occurrence of depolymerization during the frozen storage. This postulation was further supported by the fact that the scattered intensity at zero angle, *I*(0) of the SAXS data listed in Table 1, which is related to the *M_w_* of the solutes in solution [7,12], decreased with the frozen storage time. Our findings using SAXS were in agreement with those of Ribbato *et al*. [3], who found from the SDS-PAGE results that the amount of glutenin subunits of high molecular mass decreased as the frozen storage time increased, suggesting that a depolymerization took place during storage under frozen conditions, and that this depolymerization increased with time. The water redistribution and ice crystal growth during frozen storage would destroy the nonconvalent and/or covalent bonds in the gluten network, resulting in a decrease in *M_w_* [2]. As for depolymerization of gluten, some studies have showed that glutenin macropolymer was composed by weak aggregates of glutenin subunits that could be broken during the mixing of the dough, leading to the liberation of oligomers and dimers of a defined composition. Some low-molecular-weight (especially type B) and x-high-molecular-weight glutenins could be depolymerized if doughs were extensively mixed [13,14,15]. These study results implied that if the mechanical energy was high enough such as in the case of mixing and expanding during water turning into ice, the nonconvalent and/or covalent bonds of gluten would be weaken, even be broken.

**Table 1:** Structural parameters obtained from the SAXS data of different frozen time gluten.

Sample	*R_gSAXS_* (nm)	*I*(0) (a.u)	Shape *	Chi^2^
Control gluten	12.84 ± 0.38	(2.74 ± 0.14) × 10^5^	Globular	2.180
30 days frozen	9.71 ± 0.35	(2.061 ± 0.082) × 10^5^	Globular	0.7119
60 days frozen	8.24 ± 0.25	(1.705 ± 0.052) × 10^5^	Globular	1.107
90 days frozen	6.98 ± 0.19	(1.494 ± 0.041) × 10^5^	Globular	1.970
120 days frozen	6.21 ± 0.19	(1.225 ± 0.038) × 10^5^	Globular	1.311

* Shape was determined from the Kratky plot.

**Figure 2:**
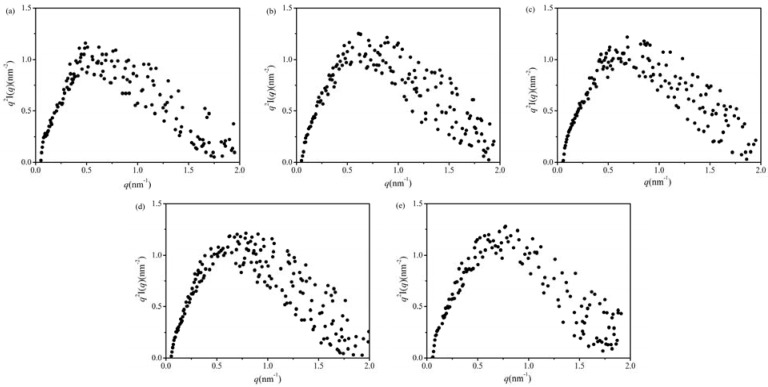
Kratky plots scattering curves from various frozen storage time states of gluten. (**a**) the control gluten. (**b**) 30-days-stored gluten. (**c**) 60-days-stored gluten. (**d**) 90-days-stored gluten. (**e**) 120-days-stored gluten.

Figure 2 shows the Kratky plots (*q* *vs*. *q*^2^*I*) of the frozen gluten with the various frozen storage time. The expression of the intensity function in the form of the Kratky plot is very useful for describe the structural characteristics of a chain molecule, and, consequently, is often used in polymer science [16].

Since an unfolded protein is thought to behave like a chain molecule, the Kratky plots should be applicable in the protein folding study [17]. For protein folding, a clear peak in the Kratky plot of the scattering curve indicates a compact globular structure [18,19], while the Krathy plot has a plateau and then increases gradually with *q* presents a folding intermediate such as a molten globule state [17,18,20], and the peak position should depend on *R_g_*. As can be seen in Figure 2, the Kratky curves for all frozen samples were similar, and the well defined peaks in the small-*q* (0.3 < *q* < 1) indicated the frozen glutens had a globular shape (also summarized in Table 1). The peak positions are shift slightly to higher *q* during the frozen storage, indicating that the *R_g(SAXS)_* value were decreased with the frozen storage time which was consistent with the *R_g(SAXS)_* measurement. This result confirmed that the gluten had quasi-spherical chain conformations and during the frozen storage the *R_g(SAXS)_* decreased with frozen time.

### 2.2. SEC-MALLS Analysis

**Figure 3:**
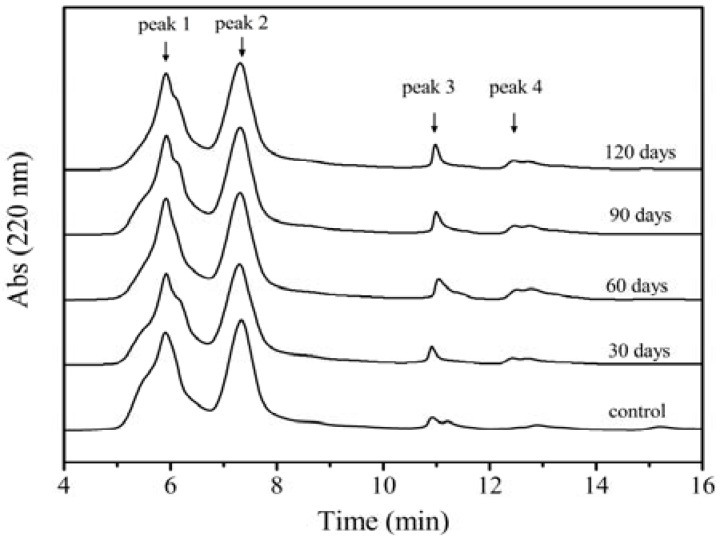
SEC profiles showing changes in gluten protein after frozen storage for different time.

Figure 3 shows the changes of frozen gluten samples with different frozen storage time in the elution profile from SEC. The profile indicates that the control gluten was composed of three fractions (peaks 1–3), while the frozen stored glutens were composed of four fractions. Fraction 1 (peak 1) and Fraction 2 (peak 2) presented the high-molecular-weight (HMW) gluten proteins, whose elution time ranges were around 5.9 min (peak 1) and 7.2 min (peak 2), respectively. The low-molecular-weight (LMW) gluten proteins were represented by peak 3 and peak 4. Data of Figure 2 clearly show that the peak area of the HMW fractions (peak 1) for the control is the highest, reaching up to 49.57%.

As the frozen storage time increased, the elution time of peaks 1 and 2 of the frozen stored samples was negatively delayed compared with that of the control gluten, implying that the average *M_w_* represented by peak 1 and peak 2 was decreasing, and their areas decreased as well. For the frozen gluten stored for 120 days, the areas of peaks 1 and 2 were reduced to 40.83% and 38.03%, respectively, while those of the control were 49.57% and 45.78%. Evidently, the loss of protein was due to the depolymerization of gluten polymeric proteins, which was attributed to the ice recrystallization and water redistribution during the storage [21,22]. Contrarily, there was little change in the elution time of the peak 3 and its area increased with the increasing storage time. A new absorption peak (peak 4) appears for all the frozen stored gluten samples. The area of the peak 4 increased with the storage time. It indicated that the longer the storage time, more LMW gluten proteins there were. The area of peak 4 for 120-day-stored gluten was three fold larger than that for 30-day-stored gluten (data not shown). The increase of LMW gluten proteins is further evidence of the depolymerization of gluten polymeric proteins during the frozen storage.

**Figure 4:**
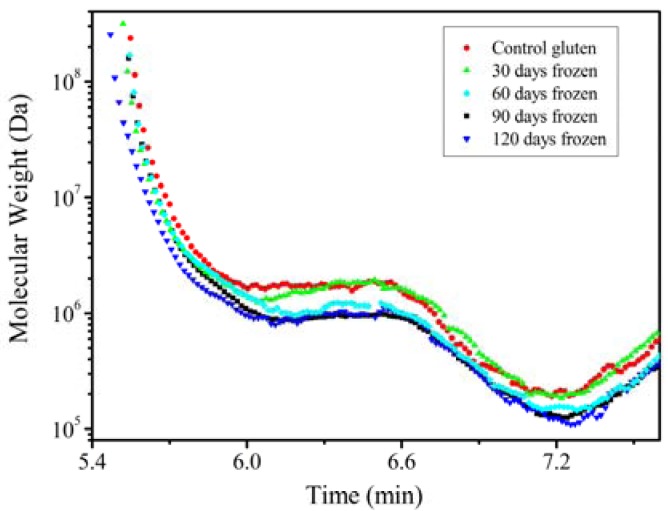
Effect of frozen storage time on the molecular weight of the wheat gluten solution.

Figure 4 shows the effect of the frozen storage time on *M_w_* of the wheat gluten. As shown in Figure 4, *M_w_* of the gluten polymer is very broad (10^5^–10^9^ Da). It is noted that after 7.2 min of elution time the *M_w_* of all of the samples increases sharply. This phenomenon contradicts the SEC-MALLS theory that *M_w_* of proteins in elution decreases with increasing elution time. The erratic upward trend in the *M_w_* *vs.* time curve has been reported. Wyatt reported that this upward swing could be due to branching, microgel components or local changes in d*n*/d*c* [23]. Bean reported that trace amounts of albumins and globulins were found in the gluten protein extract, which was eluted with the LMW gluten proteins of similar *M_w_*. The presence of albumins and globulins changed the measured value of d*n*/d*c*, leading to changes in the *M_w_* calculated by Equations (1) and (2) and the anormal upward swing in the *M_w_* curves [10]. However, the gluten protein extract undoubtedly contains the albumins and globulins; thus an advanced and elaborate purification operation is required in order to remove the albumins and globulins to a complete extent.

The *M_w_* from the elution time of 5.3–7.2 min in Figure 4 correspondd to the peaks 1 and 2 in Figure 3. It is clear that peak 1 and peak 2 consisted of HMW proteins whose *M*_w_ ranged approximately from 10^5^ Da to 10^9^ Da. The sizes for gluten proteins in peak 3 and peak 4 composed of LMW cannot be determined accurately and are not illustrated in Figure 4 due to the limitations of the MALLS detector. However, the baking quality of wheat flour is closely related with *M_w_* and size distribution and content of gluten protein polymers [11], therefore, the knowledge for the HMW fraction (peak 1 and peak 2) is adequate to understanding the dough performance.

As shown in Figure 4, with increasing frozen storage time, the *M_w_* of the gluten proteins decreased obviously, which was consistent with the SAXS conclusion. Before the 6.0 min elution time, the *M_w_* of the sample after stored for 30, 60, 90 and 120 days was obviously lower than the control where the *M_w_* ranged from 10^6^ Da to 4 × 10^8^ Da. On the other hand, between the elution times from 6.0 min to 7.2 min, except for the fact that the *M_w_* of the 30-day-stored gluten was similar with that of the control, the *M_w_* of the frozen stored gluten declined with storage time. The *M_w_* for the 120-day-stored gluten, whose *M_w_* ranged from 10^5^ Da to 3 × 10^6^ Da, was the lowest. There was some evidence from SDS gel electrophoresis measurements that depolymerization of the gluten occured during the frozen storage. Ribotta *et al.* found that there was a decrease in the amount of glutenin subunits of HMW between 88,700 and 129,100 Da using the SDS gel electrophoresis technique during frozen storage at −18 °C [3]. However, the *M_w_* they measured was much lower than the *M_w_* after 120-day storage described in our experiments. This was due to the different measurement method used for *M_w_*. The SDS gel electrophoresis technique measures the size of glutenin subunits because of the addition of mercaptoethanol. However, their conclusion was in general agreement with ours that the HMW gluten polymer depolymerized during the frozen storage.

**Figure 5:**
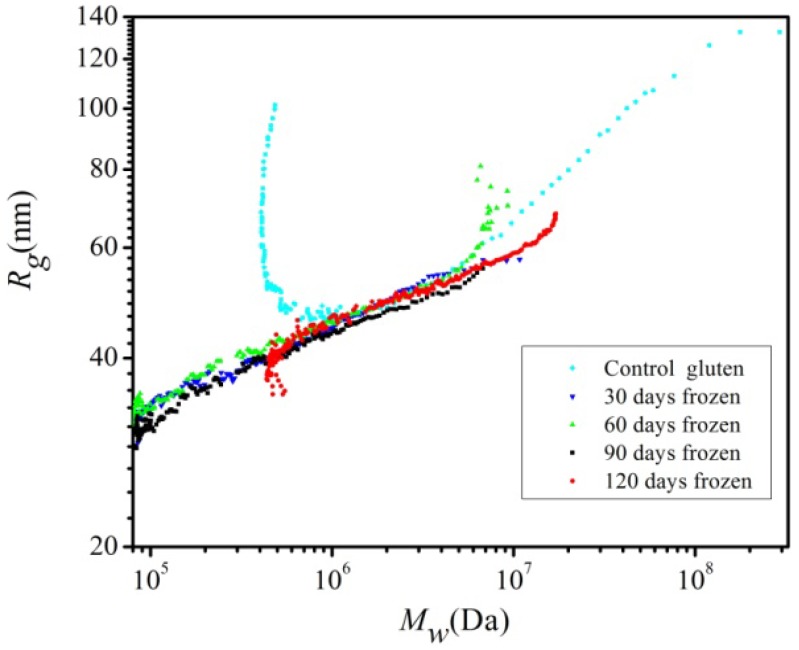
Comparison of the conformation plot, *R_g(MALLS)_* = ƒ (*M_w_*), for the control and frozen storage gluten polymeric proteins using 500 mM acetic acid solvent.

Molecular weight (*M*) and radius of gyration (*R_g_*) give conformational information of a polymer. The relation between *R_g_* and *M* is expressed as:



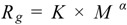
(1)


The conformation of polymer molecular chains conformation in solution can be deduced by the constant *α*. Theoretically, when *α* = 0.33, the polymer conformation is a sphere; values of 0.4 < *α* < 0.6, indicate a random coil, and when *α* = 1.0, the conformation is that of a rod. The value of *α* is calculated as the slope of the plot of ln*R_g_* *vs.* ln*M*. Figure 5 shows the comparison of the conformation plot for the control gluten polymer and the frozen samples. There were two different linear regions in the conformation plot for the control gluten which is in agreement with the findings of Carceller and Aussenac [24] and Mendichi *et al.* [11]. However, the gluten after freezing presented only one linear region. In the case of the control, the slope *α* that corresponded to the HMW region is approximately 0.28 which was close to the theoretical value (*α* = 0.33) for a compact sphere. The value of the slope *α* had no physical sense because it was lower than 0.33. Mendichi *et al.* defined this conformation as a compact quasi-sphere when studying the conformation of *Cheyenne* and *Chinese Spring* glutenin polymers with the *M_w_* above 10^6^ Da and whose *α* value was lower than 0.33 as well. The slope *a* of the control that corresponded to the LMW region was approximately 1.0; this indicates that the conformation of the untreated gluten in 500 mM acetic acid was a rod. The *a* values of the samples after frozen storage were lower than 0.33, and were 0.22, 0.15, 0.13 and 0.11 respectively for 30, 60, 90, 120-day storage. Their conformation was considered to be quasi-spherical. For all frozen stored samples, their *R_g_* tended to increase with the increase of *M_w_*, probably due to higher numbers of disulphide links. However, at constant *M_w_* the size of the frozen samples did not show a clear correlation with the storage time.

### 2.3. Effect of Storage Time on Free Amino Groups

There are only limited reports on the proteolytic enzymes associated with industrially produced vital wheat gluten [25]. However, haemoglobinase and azocaseinase activities have been observed in vital wheat gluten [26]. Redman found that 90% of gluten hydrolysing activity was effective when gluten was extracted with 0.1 M NaCl [27]. Kawamura *et al*. also suggested that gluten extracted with 200 mM acetic acid retained 85% of haemoglobinase and 40% of *N*-carbobenzoxy-L-phenylalanylalanine hydrolase activities [28]. These enzymes would hydrolyze the gluten proteins during storage, resulting in an increase of free amino group content.

**Figure 6:**
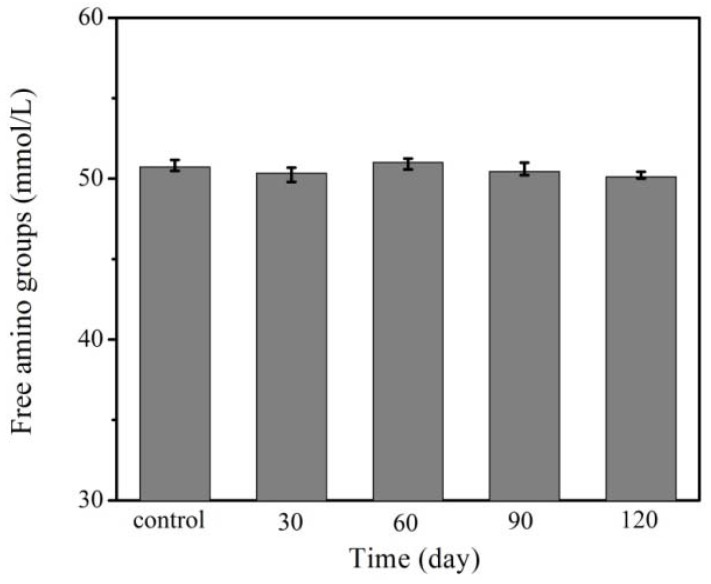
Effect of frozen storage time on the free amino group content of the wheat gluten.

In our experiment, to avoid the effect of the proteolytic enzymes, the temperature of the gluten solution preparation was kept under 5 °C and the gluten was stored at −18 °C. Figure 6 shows the effect of freeze-thaw storage time on the free amino group content of the wheat gluten. There was no a significant difference (*p* < 0.05) in the amount of free amino groups among samples stored from 0 days to 120 days. It indicated that the proteolytic enzymes were inhibited when stored at low temperature. The results of this study confirm that the observed depolymerisation is due to the frozen processing rather than the hydrolysis by proteolytic enzymes.

### 2.4. Effect of Storage Time on the Free SH Groups Content of Wheat Gluten

**Figure 7:**
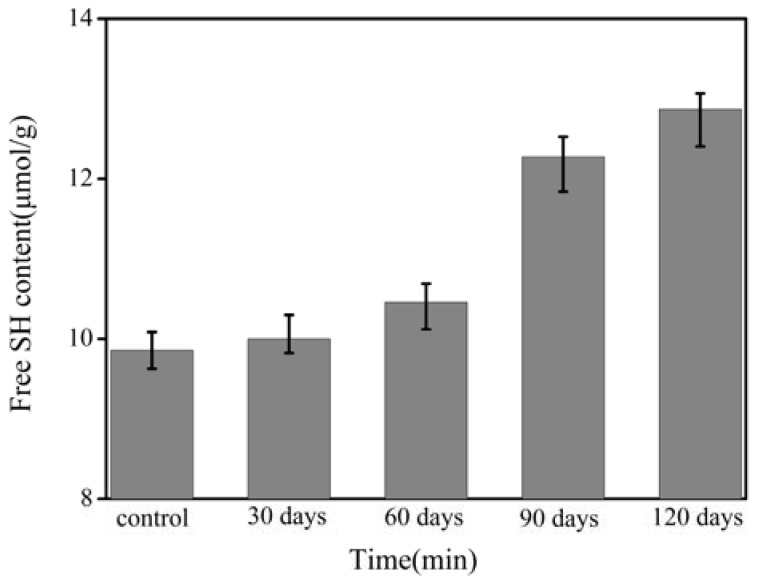
Effect of frozen storage time on the SH content of the wheat gluten.

Figure 7 shows the effect of the frozen storage time on the free SH group content of wheat gluten. It is commonly known that there is a strong correlation between the structure of gluten and the disulphide bond content. Glutenins are intra/intermolecular disulphide-linked polymers of discrete polypeptides, while gliadin is a complex mixture of largely single-chain by the intramolecular disulphide bonds [29]. Disulphide bonds play an important role in maintaining gluten structure. The breakage of intermolecular disulfide bonds could cause the depolymerization of gluten. The content of the free SH groups increases; in other words, the number of disulphide bonds was reduced. The disulphide bonds did contribute to the gluten aggregation mechanism. There is no significant (*p* > 0.05) difference in free SH content between the control and the 30-day-stored gluten (Figure 7). This finding explained why the *M_w_* determined by SEC-MALLS for the control was similar with that of the 30-day-stored gluten. It was because few disulfide bonds were broken after storage for 30 days. When the gluten was stored for 60 days to 120 days, the SH content increased remarkably with the storage time (*p* < 0.05). The free SH group content of the 90-day-stored gluten was 12.3 μmol/g, much higher than that of the 60-day-stored gluten of 10.5 μmol/g. However, as can been seen in Figure 4 the *M_w_* of the 60-day-stored gluten was approximate to that of the 90-day-stored gluten. It was suggested that the higher free SH content for the 90-day-stored gluten mainly originated from the breakage of the intramolecular disulfide bonds, which would not influence the *M_w_* of the gluten. During the frozen storage, water redistribution and ice recrystallization may be the reasons of breakage of the disulphide bonds and the depolymerization of gluten polymer.

## 3. Experimental

### 3.1. Materials

Untreated commercial wheat flour (protein content 14.2%) from Canada Hard Red Winter Wheat was purchased from Nanfang Co. Ltd (Guangzhou, China). All chemicals used were of analytical or chemical reagent grade.

### 3.2. Wheat Gluten Extraction and Freezing

Wet gluten was isolated by referring to the ICC Standard No. 137/1 (ICC 1999) with some modifications. Flour (6 g) and distilled water (3 mL) placed in the chamber of a gluten washing instrument (JJJM548, JiaDing Cereals and Oil Instruments Co. Ltd., Shanghai, China) and mixed for 1 min to form the dough. The dough was washed with sodium chloride solution (250 mL, 20 g/L) for 5 min to remove the globulin and albumin, and then with distilled water (250 mL) for 5 min to remove the residual starch and sodium chloride. Iodine solution was used to test if starch is completely removed from the washed gluten sample. Two gluten samples were washed in parallel. The hydrated gluten was centrifuged at 6,000 g for 10 min, in order to obtain the gluten with the hydration levels around 60% w/w. In the lower hydration levels case the sample was not homogeneous whereas higher hydration levels results in extensive syneresis [30]. After centrifugation, the hydrated gluten (about 4 g) was placed into cube pans with 2 cm sides and rapidly frozen in a −80 °C freezer (ULT1386–5-V39, Revco, Asheville, NC, USA) until a core temperature of −18°C was reached by the temperature probe (WS-106, Wason, Guangzhou, China). Then, the hydrated gluten was stored in a −18 ± 1 °C freezer (BCD-245, BOSCH, Stuttgart, Germany) for different times. At 0, 30, 60, 90 and 120 days, the frozen hydrated gluten was sampled and lyophilized in a freeze-dryer (Wizard 2.0, VirTis Ltd, Gardiner, NY, USA). The freeze-dried gluten was pulverized into the fine powder and screened by a 200 mesh sieve. The dry sample was stored in sealed containers until use.

### 3.3. Gluten Solution Preparation

Under continuous stirring using a vortex mixer, gluten protein samples (1.2 g) were extracted for 24 h with 500 mM acetic acid (50 mL), which is good dilute solution for gluten proteins and does not influence the structure of gluten molecular chain during dissolution according to current polymer theory. In this process, the temperature of the gluten solution was controlled not to exceed 5 °C by use of an ice bath. After extraction, extracts were centrifuged for 30 min at 12,500 g with the temperature 0 °C. The supernatant concentration determined by micro-Kjeldajhl technique (ICC stand 105/2; N × 5.7% Dm).

### 3.4. SAXS Measurement

SAXS measurements were made on solutions of gluten proteins using a SAXSess camera (Anton-Paar, Graz, Austria), which was connected to an PW3830 X-ray generator with a long fine focus sealed glass X-ray tube (PANalytical, Kassel, Germany) operated at 40 kV and 50 mA. A focusing multilayer optics and a block collimator provided an intense monochromatic primary beam (Cu-Kα, λ = 0.1542 nm). During SAXS measurement, the samples were filled into a vacuum-tight quartz capillary cell which was set in a TCS 120 temperature-controlled sample holder unit (Anton-Paar) in order to maintain the measurement temperature at 26.0 °C. The sample-to-detector distance was 261.2 mm. Each measurement was collected for 30 min. The 2D scattered intensity distribution recorded by an imaging-plate detector was read out by a Cyclone storage phosphor system (Perkin Elmer, Boston, MA, USA). The 2D data were integrated into the one-dimensional scattering function *I*(*q*) as a function of the magnitude of the scattering vector *q* (*q* = 4πsin*θ*/λ, where 2*θ* is the scattering angle). The background scattering contributions from capillary and solvent were corrected. The absolute intensity calibration was made by using water as a secondary standard. All *I*(*q*) data were normalized so as to have the uniform primary intensity at *q* = 0 for transmission calibration. The optimal concentration of proteins, suitable for data processing, was 8 mg/mL, so keep the concentration of the gluten protein solution with 8.0 ± 0.01 mg/mL.

### 3.5. SEC-MALLS Measurement

The solution of gluten (the concentration was 3 mg/mL) were separated by a HPLC system consisting of a 1,515 pump (Waters Corp., Milford, MA, USA), a vacuum degasser, a thermostated autosampler (717, Waters), a UV-detector (2478, Waters, and column compartment with a Biosep SEC-4000 column (Phenomenex, Torrance, CA, USA). The mobile phase was 500 mM acetic acid, which was vacuum filtered and degassed with 0.2-µm filters. And the samples were filtered through a 0.45 μm filter prior to being injected into the SEC-MALLS system. The SEC experimental conditions were 40 °C of column temperature, 1.0 mL/min of flow rate, 150 μL of injection volume and 220 nm of the UV-detection.

MALLS data were gathered with a multi-angle light scattering detector (DAWN HELEOSΠ, Wyatt Technology Corp., Santa Barbara, CA, USA) with 18 detection angles and a refractive index detector (DRI) (OptilabrEX, Wyatt Technology Corp) operating at 658 nm. The normalization of the voltages from the photodiodes at each scattering angle was performed by measuring the scattering intensity of a bovine serum albumin (BSA). The value of the d*n*/d*c* is 0.1767 ± 0.0028 mL/g.

### 3.6. Quantiﬁcation of Amino Groups

Changes in free amino groups were determined spectrophotometrically using *o*-phthaldialdehyde (OPA) [31,32]. OPA (40 mg) was dissolved in ethanol (1 mL). In a separate solution disodiumtetraborate decahydrate (1.905 g) and sodium dodecylsulfate (50 mg) were dissolved in distilled water (40 mL). The two solutions were mixed and volume brought to 50 mL with distilled water. This OPA reagent was stored in a dark bottle in a refrigerator. One part of 2-mercaptoethanol (ME) was mixed with 21.27 parts of the OPA reagent just before the use in the assay.

To the clear supernatant gluten (0.4 mL) the OPA reagent (8 mL) containing ME was added in a microtitre plate and absorbance read at 340 nm. The results were calculated against a serine standard curve. Four replicates were made for each determination.

### 3.7. Free Sulfhydryl (SH) Determination

Free SH groups were determined according to the produce of Veravwerbeke *et al.* [33] with minor modifications. Free SH groups were determined spectrophotometrically after reaction with 5,5′-dithio-bis(2-nitrobenzoic acid) (DTNB) [34]. Samples (1 mL of supernatant protein) were shaken for 10 min in the sample buffer (1.0 mL) composed of 0.05 M sodium phosphate buffer (pH 6.5, containing 2.0%, v/v, SDS; 3.0 M urea; and 1.0 mM tetrasodium ethylenediaminetetraacetate). DTNB reagent dissolved in 100 µL of the above mentioned buffer (0.1%, w/v) was mixed with the gluten protein solution. The absorbance of the mixture at 412 nm was read after 45 min. Absorbance values were converted to amounts of free SH using a calibration curve with reduced glutathione (0–0.10 µmol).

## 4. Conclusions

Results from this study suggest that frozen storage of the hydrated gluten at −18 °C generated decreased in *M_w_* (especially ranged from 10^5^ Da to 10^9^ Da) and *R*_g_ of the gluten. Because of the water redistribution, ice recrystallization, and the relatively strong mechanical energy during water turning into ice in frozen storage, the *M_w_* of the gluten decreased due to the break of the intermolecular disulphide bonds between the gluten polymers. The results from the SEC-MALLS also indicated that the slope of the conformation plot decreased with the storage time which deduces the conformation of the gluten changed, but as the decreased of the *M_w_* and *R*_g_ the change of the chain conformation did not show a clear correlation with the storage time at constant molecular weight. This research provides the general information on the molecule basis during the frozen storage which may relate to their role in food processing.
